# Supplementary material for: Are carbon emissions trading and green financial instruments synergistic? -Comprehensive quantitative research based on content analysis
Source: PLoS One. 2024 Mar 7;19(3):e0298601. doi: 10.1371/journal.pone.0298601 (PMC10919601; doi:10.1371/journal.pone.0298601)
Supplement: S1 Data — (ZIP) [file pone.0298601.s001.zip › Data/地级市Z_F工作报告.zip!/╣π╬≈/║╙│╪╩╨/└┤╫╘╣1⁄2╓┌║┼í╛┬φ┐╦╩2╛▌═°í┐_2014.docx]

各位代表：

现在，我代表市人民政府向大会作政府工作报告，请予审议，并请政协委员和列席会议的同志提出意见。

一、2013年工作回顾

2013年，面对经济下行压力不断加大的严峻宏观经济环境，面对有色金属市场继续严重萎缩、水电大幅度减产减发以及蔗糖价格大幅下跌等困难，市人民政府在自治区党委、政府和市委的坚强领导下，按照“稳进突围、提速发展”的总基调，以坚定决心和强力措施，稳步推进产业增量提质、民生保障提升、开发扶贫攻坚、城乡新貌新风和执行力提升“五大工程”。在极为艰难的条件下，全市经济实现了恢复增长，各项事业取得了新成果。预计，全年地区生产总值518亿元，增长6%；财政收入50.23亿元，增长12.74%；全社会固定资产投资340亿元，增长34%；规模以上工业总产值350亿元、增加值125亿元，分别增长19%、9%；社会消费品零售总额198亿元，增长12%；城镇居民人均可支配收入19760元，增长10%；农村居民人均纯收入5220元，增长13%；城镇登记失业率2.82%；居民消费价格指数涨幅控制在2%以内。

一年来，我们突出抓好以下重点工作：

（一）聚力调整产业结构。坚持转方式、调结构，激活存量、做大增量，推动三次产业协调发展。

深入实施环境倒逼机制，推动有色金属行业整合提升，新建大任产业园区，拓展现代工业发展新空间，入园二级公路、园区路网工程和7家企业“出城入园”项目开工建设。充分发挥市国投公司国有资本运营平台和融资平台作用，推动经营性国有资产集中统一经营和监管。大力发展循环经济，培育养生健康产业，推动新兴产业提质发展，酿酒、优质饮用水、医药制造产业产值分别增长86%、80%、30%，酿酒、优质饮用水产业产能、总产值均排全区首位。南方集团、泰星公司分别被认定为国家高新技术企业、广西创新型企业。河池·南丹工业园区总产值突破100亿元，成为我市首个百亿元园区。广西茧丝检测中心建成使用。全年发展微型企业1931家。新增国家地理标志保护产品1个、广西著名商标9件、广西名牌产品4个。

“三农”工作稳中提质。落实最严格的耕地保护制度和占补平衡制度，连续15年实现耕地占补平衡。粮食生产保持稳定，全市粮食作物播种面积415.77万亩，总产量103万吨。推行特早熟、特晚熟、特优质，错季生产、错峰上市、错位发展“三特三错”农业发展新模式，增量提质特色优势农业。新种桑园3.73万亩，总面积达76.35万亩，巩固了广西桑蚕产业第一大市地位；牛、山羊饲养量和出栏量及内陆大水面养殖面积保持全区第一。创新农业生产组织方式，新增农民合作社203个。创建农业部水产健康养殖示范场3家。获得认定自治区级农业产业化龙头企业8家。农作物耕种收综合机械化率提高7.32个百分点，达37.6%。林业总产值超110亿元，提前两年完成“十二五”规划目标。治理水土流失29平方公里，新增恢复改善灌溉面积47.62万亩，解决37万人饮水不安全问题。

以巴马长寿养生国际旅游区成为广西三大国际旅游目的地之一为契机，组建旅游区管理机构，编制盘阳河流域养生长寿健康产业规划、旅游区建设行动计划和旅游区基础设施建设大会战行动计划，推动旅游业快速发展。全年接待游客1280万人次，增长20%；旅游总收入112亿元，增长25%。文化产业加快发展，建成壮乡英雄文化园等项目，成立河池市旅游文化商品研发中心，宜州、东兰、都安成为自治区首批特色文化产业示范县（市）。东兰县、凤山县荣获“中国长寿之乡”称号。铜鼓山歌艺术节影响力进一步扩大，河池被评为“中国最具魅力节庆城市（地区）”。加快建设各类专业市场，带动商贸业提质发展。通信和交通运输等其他服务业稳健发展。

（二）加快项目投资建设。实行市、县两级四家班子领导联系重大项目制度，加快重大项目建设，全市全社会固定资产投资增速排全区前列。投资结构进一步优化，基础设施、民生、社会事业等领域投资比例继续提高。全市公路水运交通项目完成投资37.95亿元。建成河池金城江机场，已完成校飞具备通航条件。河池至都安高速公路主体工程基本完工。争取到农村公路项目资金7.79亿元，建设通建制村水泥路221条1191.3公里。凤糖罗城公司技改扩建、博冠纸业公司年产5万吨漂白蔗渣浆等一批重点项目竣工投产。

（三）提升城乡建设品质。抓住“柳来河一体化”规划实施新机遇，积极筹划宜州新区建设，推动金城江—宜州同城化发展。市中心城区南桥拓宽工程、建设路改造工程等一批项目竣工。扎实推进城镇扩容、新区建设、旧城改造升级和基础设施建设。开工建设36个村镇建设提质试点工程，实施43个城区风貌改造、特色名镇名村试点项目。以规划建村、环卫进村、民主治村、新风树村、干部入村、创建名村“六村”为抓手，大力开展“美丽河池·清洁乡村”活动，取得阶段性成效，“党领民办、群众自治”的清洁乡村工作模式在全区推广。市中心城区“两违”养殖场整治、在建工地防尘污染设施配建基本完成，建材、小商品、夜市等专业市场建成使用。

（四）狠抓开发扶贫攻坚。多方筹措扶贫资金，创建了一批产业扶贫示范典型，培育了一批产业扶贫龙头企业，打造了一批农业产业品牌。香猪、种草养畜、水面渔业等一批扶贫养殖产业年产值超过20亿元。2012-2013年冬春新种核桃56.76万亩，是前10年总面积的4倍。创新环江大安“整乡推进”扶贫新模式，深入实施乡村旅游开发扶贫“十百千”示范工程，金城江区英洞瀑布山庄成为广西四星级乡村旅游区。大化岩滩库区处遗规划攻坚战取得阶段性成果。整市推进扶贫产业、“整乡推进”开发扶贫模式得到国务院扶贫办的肯定。年内全市农村贫困人口减少20万人以上。

（五）推进生态文明建设。推行环境污染第三方治理模式，委托专业公司，引进先进技术，加快重金属污染综合防治和矿山环境修复。争取国家专项资金2200万元，实施龙岩滩水库生态环境保护试点项目。环江“中国南方喀斯特”世界自然遗产提名地通过世界自然保护联盟专家实地考察评估。南丹县获得全国“国土资源节约集约模范县”称号。投入3028万元治理地质灾害隐患点126处。全市环境质量持续好转，市中心城区空气质量优良率达97.5%，主要河流监测断面、城市集中式饮用水源地下水、城区饮用水水质达标率100%。治理石漠化面积264平方公里，森林覆盖率提高0.27个百分点，达68.19%。

（六）强化财税金融管理。深入推进财源建设，实现财政收入稳步增长，公共财政预算收入26.98亿元，增长21.68%。加强财政支出预算执行管理，保证社会事业和民生领域等重点支出，公共财政预算支出193.34亿元，增长9.89%，其中民生支出占80%左右。本外币存款余额730.09亿元，比年初增加101.71亿元，增长16.19 %；贷款余额419.53亿元，比年初增加55.3亿元，增长15.18%。成功发行河池首支企业债券11亿元。信贷资金提升了金融服务经济社会发展的质效。

（七）深化改革开放合作。成功举办闽商河池行、港商河池行、侨商广西行—柳来河区域一体化建设（河池）推介会等多项招商引资活动。在第十届中国—东盟博览会上，我市签约项目27个，总投资161.65亿元，已开工13个，到位资金7.28亿元。全年实施招商引资项目（含续建）520个，总投资1223.64亿元，实际到位内资230亿元，增长13.86%，全口径利用外资7137万美元。邀请“两岸四地”书画摄影家走进河池宣传河池。与泰国罗勇市缔结为友好城市，实现了我市国际友城零的突破。加入湘桂黔毗邻地区经济技术协作区。启动实施“柳来河一体化”发展规划。推进左右江革命老区振兴规划编制。

（八）倾力发展社会事业。10项惠民工程全面完成。筹集1.42亿元发展学前教育，逐步缓解“入园难”、“入园贵”问题；义务教育均衡发展，投入1.3亿元建设农村学校食堂，营养改善计划受益学生23.3万人，市职教中心学校新校区建成启用。新增城镇就业2.82万人，农村劳动力转移就业9.3万人。大化获评全国科技进步先进县。新增全国重点文物保护单位2处，罗城乡镇文化站规范管理成为全国示范。全民健身活动蓬勃开展，体育事业不断推进。社会保障体系进一步完善，“五险”参保人数110.61万人。城乡居民最低生活保障制度和医疗救助制度进一步健全。出台《河池市老年人优待规定》，在广西率先建立高龄老人补贴制度。人口计生工作保持全区领先水平。全面完成保障性住房建设和农村危房改造任务。成功承办韦国清诞辰100周年系列纪念活动。

（九）推动社会治理创新。建立健全社会稳定风险评估机制，深入开展领导干部大接访活动，抓好关闭的有色金属企业维稳工作，妥善处置原红茂矿务局、原罗城矿务局、库区移民和其他国企改制等遗留问题，成立河池市仲裁委员会，进一步拓宽矛盾纠纷化解渠道。积极开展“打盗抢、保民安”和“扫黄打非”专项行动，确保社会大局稳定。都安“一村一警”警务模式、天峨“云榜模式”成为广西乃至全国农村基层社会治安管理的示范。成立劳动人事仲裁委员会，在全区率先实现市县两级劳动争议仲裁院全覆盖。强化安全生产管理，全市安全生产形势持续好转。全年社会公众安全感排全区前列。

（十）加强政府自身建设。认真贯彻落实中央“八项规定”，努力解决“四风”问题，推动权力公开透明运行，推动简政放权、转变职能和改进作风。实行市政府常务会议无纸化，全市性会议比上年减少36%，节约开支320多万元。建立“市长企业接待日”制度，多形式、多渠道解决企业发展中遇到的困难和问题。在全区率先启动运行公共资源交易中心，率先完成食品药品监管体制改革。取消和调整行政审批项目66项，政务服务群众满意率99.9%。深入开展网络问政工作。市政府被命名为自治区级第二批依法行政示范点。市物价局荣获广西唯一“全国物价工作先进集体”称号。我市软环境满意度全区排位从2011年的第13位上升到2013年的第11位，政务环境从第12位上升到第6位，党风廉政建设满意度从12位上升到第4位。

一年来，民族事业全面进步。依法治市建设稳步推进。国防教育、国防后备力量建设不断加强，双拥共建工作进一步深化。统计、审计、监察、编制、法制、侨务、宗教、外事、人防、机关事务、档案、地方志、防震减灾、粮食、供销等工作迈出新步伐，妇女儿童、老龄、残疾人保障等事业取得新进展，中直、区直驻河池各单位取得新成绩，多个单位、部门荣获全国或自治区级荣誉。

各位代表，一年来的成绩来之不易。虽然增长低于预期，增速有所放缓，但质量提高，效益好转，布局、结构和环境优化，为调结构、促改革、强后劲打下了坚实基础，我市经济社会发展是健康协调可持续的。这凝聚着广大干部群众的心血和汗水，汇集了全市人民的智慧和力量。在此，我谨代表市人民政府，向全市各族人民，向人大代表、政协委员，向各民主党派、工商联和社会各界人士，向驻河池中直、区直各单位和驻河池部队，向所有关心支持河池发展的朋友们，致以崇高的敬意和衷心的感谢！

一年来，我们深深体会到：必须坚持处理好追赶与转型的关系，做到加快速度、扩大总量和调整结构、转变方式相互促进、实现双赢。必须坚持处理好做强产业与保护环境的关系，做到发展实体经济，壮大特色产业和重视环境保护，建设生态文明相互促进、实现双赢。必须坚持处理好市场调节与政府调控的关系，做到发挥市场在资源配置中的决定性作用和搞好市场监管、社会服务相互促进、实现双赢。必须坚持处理好经济发展与社会和谐的关系，做到发展经济改善民生和营造稳定社会环境相互促进、实现双赢。必须坚持处理好借助外力与自身发力的关系，做到向上争取支持、向外引进要素和向内激发活力、向下抓好落实相互促进、实现双赢。

同时，我们也清醒地看到，当前我市经济社会发展中还存在许多困难和问题：经济基础薄弱、产业转型升级难度大、重大项目不多、经济增长内劲不足、扶贫任务异常艰巨；发展潜力未能完全发挥，配套基础设施滞后，资源开发程度较低；国企改革和库区移民遗留问题较多，维稳形势依然严峻；发展环境仍需改善，少数公务员服务意识和依法行政观念不强，行政效能有待提高。对这些困难和问题，我们正采取有力措施，尽最大努力加以解决。

二、2014年工作安排

今年是全面贯彻落实十八届三中全会精神、全面深化改革的开局之年，也是实施“十二五”规划的攻坚之年。做好政府工作，任务繁重而艰巨。

政府工作总体要求是：深入贯彻落实党的十八大和十八届三中全会精神，坚持稳进突围、提质发展总基调，把改革创新、扩大开放贯穿经济社会发展各领域各环节，突出开展党的群众路线教育实践活动和“五大工程深化年”活动“两个抓手”，推动工业发展、农业产业建设、交通基础设施建设、巴马长寿养生国际旅游区建设、城乡建设、干部作风转变“六个突围”，加快建设幸福新河池，追赶全国全区同步建成小康社会。

经济社会发展主要预期目标是：地区生产总值增长8%，财政收入增长10%，规模以上工业增加值增长15%，全社会固定资产投资增长18%，社会消费品零售总额增长11%，外贸进出口总额增长10%，招商引资增长8%，城镇居民人均可支配收入增长10%，农村居民人均纯收入增长13%，城镇登记失业率控制在5%以内，居民消费价格指数涨幅控制在4%以内，人口自然增长率控制在8‰以内，节能减排指标控制在自治区下达的目标范围内。

要实现上述目标，必须紧紧抓住各种新机遇。当前，国家继续实施稳增长、调结构、促改革、惠民生的政策，中央支持广西打造新的战略支点，支持珠江—西江经济带上升为国家战略；自治区加快“柳来河一体化”发展，加快滇桂黔石漠化综合治理，推进左右江革命老区振兴规划；我市巴马长寿养生国际旅游区建设全面加快，实施环境倒逼机制已见成效，实施“五大工程”建设已有效果，拓展城市发展空间已成共识，这些内外条件，将使我市的资源、生态、政策优势更加明显。我们既要抓住机遇，坚定信心，又要迎接挑战，克难奋进。

为确保实现今年的目标任务，必须深入开展“五大工程深化年”活动，重点抓好以下工作：

（一）调整优化工业产业结构，推动工业提质发展

加快产业园区建设。继续推进大任产业园区和河池·都安临港工业园区基础设施建设，年内完成配套基础设施建设投资8.5亿元以上，“出城入园”企业达11家以上。整合提升现有市级园区和金城江工业集中区，大力支持县级工业园区加快建设。全市工业园区（集中区）工业总产值实现230亿元以上，其中，河池·南丹工业园区总产值达110亿元以上。

推进生态环保型有色金属产业示范基地建设。继续实施《广西河池生态环保型有色金属产业示范基地规划》及其《方案》，提升有色金属产业，年内有色金属企业整合到30家左右。加快建设南方集团10万吨铅锑联合冶炼、成源公司技改升级、鑫锋蓄电池、生富公司搬迁技改等一批重点项目。全年有色金属产业产值实现200亿元以上。

加快特色优势产业发展。深度开发水电，推动热电联产项目落地，谋划风力发电项目。打造以广维公司为龙头的化工产业园，推进河化公司产品结构调整及能量系统优化建设。加快西江·鱼峰水泥项目建设，推动碳酸钙产业发展。推进恒业公司印染项目开工建设，尽快形成从种桑、养蚕到印染、服装完整产业链。推进丹泉名酒文化城、天龙泉酒业黄酒和白酒技改扩建、德胜酒业清酒技改建设，打造“白、红、黄、清”并进发展、特色突出的全国酒业基地和产业集群。做大巴马丽琅、九千万等知名饮用水品牌，引进大企业集团，整合提升优质饮用水产业。整合糖蜜、蔗渣资源，推进宜州华美集团高级生活用纸等项目建设，促进机制糖产品结构升级。积极培育新材料、新能源、生物医药、长寿养生、节能环保等战略新兴产业。电力、化工、茧丝绸、酿酒、优质饮用水产业产值分别达80亿元、24亿元、25亿元、40亿元、10亿元。

加大服务企业力度。完善企业科技创新服务平台，年内建成自治区级研发中心、企业技术中心各1家，实施科技创新项目10个以上。深入实施“抓大壮小扶微”工程，坚持“市长企业接待日”制度，利用河池市企业服务中心，为企业发展提供优质服务。实施强优企业培育计划，发展超50亿元企业1家以上，10亿元企业8家以上，新增规模以上企业8家以上。

大力发展非公经济。进一步优化服务，探索通过投融资服务平台和“河池非公企业之声”服务平台，为非公企业发展提供优质服务，充分激发非公经济的活力和创造力，推动非公经济跨越发展。年内全市非公有制资本投资规模增长25%以上，新增6万个就业岗位。

（二）加快发展特色效益农业，推进农业产业化

做大做优特色产业。实施“粮安工程”，稳定粮食种植414万亩，总产量102万吨。继续推行“三特三错”农业发展模式，因地制宜发展各地农业产业化，全面提升优势农业产业上规模、提品质、增效益。新增桑园1.6万亩以上，巩固全区第一位置。新增核桃80万亩、油茶1.7万亩、烤烟0.9万亩、毛葡萄2.5万亩、水果2万亩以上。糖料蔗种植稳定在120万亩以上。栽培食用菌1.5亿棒，产值达5亿元以上。继续实施“百万林农千元增收”工程，做大香猪、菜牛、山羊等产业。大力发展库区大水面养殖。

提高农业产业化水平。加快农业和农村基础设施建设。支持农业龙头企业做大做强，培育年销售收入5000万以上农业产业化龙头企业40家以上。新增农民合作社100个以上。发展适度规模经营，全市土地流转30万亩以上。培育壮大特色农业品牌，年内每个县（市、区）新增培育名特优农产品品牌2个以上、涉农注册商标3个以上。继续落实农机具购置补贴政策，加大基层农机推广体系建设，不断提高农业机械化水平。

（三）大力发展第三产业，培育新的经济增长点

推动旅游业更好更快发展。启动实施巴马国际旅游区基础设施建设大会战，加快道路交通、生态环保、旅游服务等基础设施建设。坚持“政府引导、市场开发、农户参与”原则，统筹推进国际旅游区建设与旅游开发扶贫、新农村建设相结合，同步提速提质丹金宜罗环旅游业发展。全年接待游客人数增长17%以上，旅游收入增长18%以上。办好河池铜鼓山歌艺术节和“一县一节”文化旅游活动。完成社会文化固定资产投资1.5亿元，文化休闲娱乐、广播影视、出版物营业收入突破4亿元。

加快商贸物流业发展。完善市中心城区专业市场建设，引导各类经营归行入市。各县（市、区）结合资源优势和特色，建设和发展一批区域性大型专业交易市场。加快发展第三方物流，重点发展汽车、冶金等生产性专业物流，促进制造业与物流业联动发展。扩大有色金属原料进口和产品出口，推动优质饮用水、茧丝绸、长寿食品等产品出口。新增进出口企业4家。

促进城乡居民扩大消费。努力提高城乡居民收入，增强消费能力。积极培育旅游、文化、健身、娱乐、养生等新的消费热点。认真组织好重点消费品的集中促销活动。继续实施以“万村千乡市场工程”为载体的农村商品零售网络建设，年内完成7个乡镇商贸中心、5个物流配送中心和12个农贸市场改造建设。保持房地产平稳健康发展，完成投资22亿元以上。继续做好市场保障和价格稳定工作，整顿和规范市场价格秩序。

（四）着力抓好重点项目建设，保持固定资产投资增长

推进重大交通项目建设。坚持“抓好铁（路）公（路）机（场）、打通金水道”，加快构建内外通道便捷、畅通、高效、安全的现代综合交通运输体系。铁路方面，年内竣工使用罗城（牛毕）火车站，全力推进河池至南宁客运专线、黔桂铁路扩能改造项目前期工作。公路方面，建成河池至都安高速公路等项目，开工河池至百色高速公路，推进河池至桂林、贺州至巴马、田阳过巴马至凤山（三门海）高速公路前期工作。加快建设三岔至宜州、南丹吾隘至东兰、巴马至平果（坡造）、凤山至凌云（逻楼）、天峨至凤山（凤山段）等二级公路，续建大化至巴马、都安至武鸣、罗城至柳城（六塘）、环江至金城江（东江）等二级公路，力争开工罗城至融水（洞头）、凤山至乐业新化二级公路。扎实推进5条约120公里重要县乡连网公路改造项目前期工作，力争年内实现开工。加快农村公路建设，按要求完成全年通村水泥路建设任务。加强农村公路管理，逐步实现农村公路养护管理正规化、标准化、科学化。机场方面，完成河池金城江机场后续工作，确保年内通航；推动河池巴马通用机场前期工作。红水河复航方面，续建都安港红渡作业区技改项目；力争开工西南水运出海中线通道红水河龙滩翻坝系统一期工程；加快推进河池港大化、南丹作业区和岩滩水电站过船设施扩建项目前期工作。

抓好其他重大项目建设。重点抓好16个自治区统筹推进和220个市级统筹推进的重大项目建设，年内完成全社会固定资产投资401亿元。加快推进县县通天然气工程、罗城100万吨水煤浆等项目，竣工都安那精煤矿年产100万吨煤、岩滩水电站扩容工程、南丹拉纳懂托水电站等项目。全年实施重大工业项目27个，总投资81.09亿元，年度计划投资28亿元。加快推进巴马华昱百魔洞养生度假区、都安三岛湾等一批重大旅游项目和一批重大民生工程项目建设。

全力做好协调服务。落实重点项目领导联系制度，推行“六个一”项目管理模式等机制，简化环节，优化流程，创新方式，缩短时间，提高效率，加快项目建设进度。发挥各职能部门作用，帮助解决项目用地、审批、资金等突出问题，促进更多项目开工建设和竣工投产。

（五）完善城市功能，加快新型城镇化发展

推进以人为核心的新型城镇化，加快推进市中心城区建设，带动县（市）城区、乡村集镇突围发展，城镇化率提高1个百分点以上。

抓好市中心城区建设。提速城东、城西片区建设，竣工城东片区南北面路网、水电广场等项目，加快推进芝田路网工程等在建项目。提质旧城改造，疏通老城路网，重点抓好江北西路重建、江北东路改造、建设路延长线改造、水厂码头沿江路贯通工程等项目。开工建设东江氮肥厂和河池化工总厂棚户区改造等项目。市中心城区新增建成区1平方公里。以创建国家卫生城市和广西园林城市为目标，大力实施绿化、美化工程，实现绿化覆盖率34%、绿地率30%、人均公共绿地7平方米。完善宜州新区相关规划，稳步推进宜州新区建设。

抓好特色城镇建设。打破行政区划界限，着力实施大县城战略，主动融入桂中城镇群，积极推进县（市）改区、乡改镇、乡镇改街道。各县（市、区）要以“建新区、抓规划、改旧城、显特色”为重点，大力推进城区、乡镇、村屯以及县际主要交通沿线、旅游景点周边等层面的风貌塑造。启动11个名镇（乡）、139个名村试点创建工作。全面完成30个乡镇总体规划和15个建制镇控制性详细规划编制工作。引导和推动全市人均耕地不足0.3亩的30万农民入城就业创业。

深入推进“美丽河池·清洁乡村”活动。以“六村”为抓手，推广“党领民办、群众自治”的清洁乡村工作模式。每个县（市、区）新增1个以上“清洁家园”示范村和4个以上“清洁田园”示范点建设。实现以县城为中心20公里范围内的农村垃圾“村收镇运县处理”全覆盖。基本完成500人以上的自然村庄饮用水水源地保护。

强化城乡秩序治理。加大“两违”查处力度。严整市容市貌，推进市中心城区粉尘污染、交通综合、环境卫生、规范养殖、占道经营、户外广告、饮食摊点排污、乱搭乱建、文明殡葬等9个专项整治，进一步提升城市品位。严处建设乱象，加大建筑市场管理和工程质量安全执法，促进城乡面貌明显改观。

（六）发展扶贫产业，深入推进开发扶贫攻坚工程

持续打好扶贫产业和基础设施建设持久战、攻坚战。力争年内减少贫困人口20万人以上，全市贫困人口减少到80万人以下，提前一年完成《滇桂黔石漠化片区区域发展与扶贫攻坚规划（2011-2020 年)》确定的“十二五”贫困人口数量减半目标。

加快扶贫产业发展。推广环江大安“整乡推进”扶贫新模式，每个县（市、区）抓好1个开发扶贫“整乡推进”示范点，重点推动产业扶持贫困户全覆盖。大力发展核桃等八大扶贫产业，着重抓好贫困村扶贫产业种植、养殖示范基地建设，力争建成“县有万亩、乡有千亩、村有百亩”高产高效特色产业示范基地，推动形成“一县一产业”、“一乡（村）一品”特色突出的产业发展格局，确保每户贫困农户有1项以上增收项目。

夯实开发扶贫基石。进一步整合各类资金加大投入农村道路建设，实现90%行政村通四级以上公路，85%以上行政村通沥青（水泥）路，70%以上自然屯（20户以上）通屯级路。加强农村水利建设，投资6亿元实施农村饮水安全工程866处，加快一批水库除险加固、小流域综合治理、农业综合开发水土流失综合治理等工程。投资3亿元实施金城江、大化、宜州、罗城、环江、东兰等6县（市、区）农村电网改造升级工程。

多渠道增加农民收入。落实培训补贴政策，扎实开展农村劳动力职业技能、“阳光工程”、“雨露计划”、渔民转产转业、水库移民教育等培训，提高农民的科技致富能力、转移就业能力和自主发展能力。年内完成农村实用技术培训5万人次以上。深入实施农民收入倍增计划，千方百计提高农民家庭经营收入，增加农民工资性收入、转移性收入和财产性收入。

强力推进连片开发扶贫。实施《滇桂黔石漠化区域发展与扶贫攻坚河池片区实施规划（2011-2015年）》，加快推进436个贫困村“整村推进”开发扶贫。深入实施乡村旅游开发扶贫“十百千”示范工程，让更多农民受益。推进岩滩库区处遗规划攻坚战，实施水库移民基础设施项目40个。

（七）加强节能减排和生态环境保护，促进可持续发展

加强巴马长寿养生资源保护。积极配合自治区加快出台《广西壮族自治区盘阳河流域生态环境保护条例》，着手研究起草《红水河开发保护条例》，推进盘阳河流域环境整治与综合治理，保护好国际旅游区珍稀资源。加强保护景区景点生态环境，严禁破坏性开发建设，坚决遏制“两违”。做好地域、地名和特有物产注册保护，旅游区每个县完成注册或认证2-5个。

抓好生态环境建设。抓好造林绿化，稳步提高森林覆盖率。加强饮用水源地、水资源、生物多样性保护。推进市县两级土地利用总体规划中期评估和土地调查成果应用工作，实施整村推进土地整治工程，切实保护耕地，节约集约用地。推进矿产资源整合开发，严厉打击乱采乱挖、无证开采、破坏矿产资源等违法行为。加强地质环境保护和地质灾害防治。认真实施主体功能区规划，全面推进生态市、生态县建设，积极创建全国环境优美乡镇。

扎实抓好节能减排。严格落实节能减排工作责任制，深入开展环保专项行动，加强污染源在线监控和监督性检测，促使污染源全面达标排放。抓好重点企业节能降耗，淘汰落后产能，防止低水平重复建设，完成自治区下达的节能减排任务。

加强污染治理和生态修复。继续实施《广西河池市重金属污染防治规划》，强化环保目标责任制管理，推进金城江、环江、南丹3个重点防控区和宜州、罗城的重金属污染治理项目建设。用好国家资金实施龙岩滩水库生态环境保护试点项目。

（八）认真抓好财税工作，促进财政金融平稳运行

强化财力保障。全面加强征管、培育和壮大财源，优化财政支出结构，把有限的财力用于改善民生和转型升级，促进提质发展。加强沟通协作，积极引导金融机构强化“银政互动”、“银商企合作”，不断优化信贷投放结构，加大在重点产业、重点领域的金融支持力度，缓解中小企业融资难问题。年内新增贷款40亿元以上。

强化政策保障。全面贯彻落实国家税制改革、结构性减税政策以及促进西部大开发等税收优惠政策，用好用足用活有关政策，为企业发展“减负”，为经济发展“加速”。继续抓好“营改增”等工作。

强化信息保障。整合优化力量，精心做好经济运行数据的统计、分析工作，强化预测、监测，及时对倾向性、苗头性问题组织专题调研，跟踪掌握各主要行业的主要经济指标走势，形成判断准确、分析透彻、观点鲜明、建议可行的决策参考。

（九）全面深化改革开放，增强发展的动力和活力

强化治贫、治乱、治政“三治”理念， 推动我市全面深化改革取得新突破。

全面推进各项改革。推进经济体制改革。发挥市场在资源配置中的决定性作用，积极发展混合所有制经济。深化国有企业改革，组建资源性国有资产管控平台，建立经营性国有资产集中统一监管制度。深化工业园区管理改革，理顺园区管理体制和投入运行机制。完善非公有制经济发展配套措施和实施细则。深化财税体制改革，改善市县产业共建共享利益分配。深化投融资体制改革，鼓励和推动企业上市。深化农村改革。推动农村产权制度改革，发展多种形式规模经营。开展农村土地承包权确权试点，积极稳妥有序推进土地集中流转。深化社会治理体制改革。完善社会稳定风险评估办法和运行机制。把涉法涉诉治理纳入法制轨道，建立健全信访工作终结机制。健全城市综合管理标准化体系，全面提升城市管理水平。深化行政体制改革。理清行政审批事项管理权限，扎实做好“接、放、管”工作。积极推行“阳光审批”和“限时审批”制度，建立多部门联席审批机制。深化生态文明建设体制改革。创新生态环境保护机制，落实主体功能区定位。健全生态文明建设长效机制，推动矿产资源管理体制改革。从今年开始对“东巴凤”地区实行差别化考核。健全基层群众自治机制。统筹文化、科技、教育、收入分配、社会保障、医药卫生等领域改革。

提高开放合作水平。扩大开放领域，优化开放环境，积极融入中国—东盟自由贸易区、泛珠三角经济区和北部湾经济区的开放开发，提高开放水平。深化与央企、民企和自治区重点企业合作，落实与闽商、港商、侨商等商会达成的协议，引进一批对经济发展支撑力强的大项目、大企业。完成招商引资内资到位230亿元，外资到位7565万美元。优化进出口产品结构，拓展东盟等新兴市场，完成外贸进出口总额4.51亿美元。

（十）着力改善民生，扎实推进和谐社会建设

完善社会保障惠民。深入实施居民收入倍增计划，努力实现居民收入增长和经济发展同步。持续稳定和扩大就业，开展创业培训1200人，新增城镇就业2.3万人、农村劳动力转移就业7.3万人。深化社会保障体系建设，加大社会保险扩面征缴力度，实现“五险”参保人数108.65万人。新型农村和城镇居民社会养老保险参保人数139.43万人，养老金发放率100%。发挥劳动关系三方机制作用，加大劳动保障监察执法力度。落实被征地农民养老保险和生活保障。扎实开展灾害应急救助和灾后恢复重建工作。落实水库移民口粮补助调整政策，保障移民基本生活。加强城乡养老服务机构建设。发展社会福利事业和慈善事业。落实重点优抚对象抚恤补助标准自然增长机制。加强残疾人社会保障和社会服务体系建设。

发展社会事业利民。优先发展教育事业，继续实施市中心城区学校布局调整“3322工程”，完成澳门城小学、城西幼儿园主体工程、金城江区二中新校区、市实验小学的建设（维修、扩建）任务，九年义务教育巩固率达73%，高中阶段毛入学率达83%。支持名校提升建设，创建自治区级示范幼儿园1所、市级5所，市职教中心学校通过国家中等职业教育改革示范性学校验收。拓展学生资助范围，资助贫困学生25.3万人。发挥河池学院、广西现代职业技术学院优势，培养一批实用型人才。开展首届“刘三姐文学艺术奖”评奖活动。深化文体公共服务，积极开展形式多样、内容丰富的群众性文体活动和赛事。深化医疗卫生保障体系建设，新农合信息化管理实现全市“一卡通”，参合率稳固在96%以上；基本公共卫生服务经费年人均达30元；健全医疗卫生服务网络，扩大县级公立医院综合改革覆盖面。深入实施防艾攻坚。提升人口计生服务质量，持续稳定低生育水平。加快推进民族团结进步模范市建设，继续抓好扶持人口较少民族发展项目建设，支持办好罗城仫佬族自治县以及全市9个民族乡成立30周年庆祝活动。加强国防动员和民兵预备役建设，扎实做好创建全国双拥模范城市工作。

创新社会治理安民。深入开展“平安河池”建设，全面推广天峨“云榜模式”、都安“一村一警”警务模式，加强农村社会治理创新，建立矛盾化解新机制。实施“天网工程”，继续开展“打盗抢、保民安”和“扫黄打非”专项行动，严打“黄赌毒”、网络犯罪等违法犯罪行为。切实抓好村、社区“两委”换届选举工作。落实安全生产党政同责和“一岗双责”制度，持续开展安全生产大检查，深化安全专项整治，杜绝重特大安全生产事故发生。坚持食品药品科学监管理念，健全监管体制机制，创新监管方法，强化示范工程建设，严打制假售劣行为，严防食品药品安全事故发生，确保公众食品药品安全。加强公共应急管理和应急救援体系建设，不断提高突发公共事件处置能力。

实施十项惠民工程。1.社保惠民。提高城乡低保补助标准，城乡低保对象月人均补差水平分别达205元和84元以上。开工建设市老年公寓综合楼，新建五保村126个，新增农村养老机构床位1000张以上、农村幸福院14个。加强农房政策性保险工作。2.卫生惠民。加快标准化县、乡村医疗卫生服务体系建设，建设中央投资的县级医院项目1个、重大疾病防控体系项目5个，完成乡镇卫生院项目21个、社区卫生服务中心项目2个、村卫生室225个。3.教育惠民。新建乡镇中心幼儿园15所，利用农村闲置校舍改建幼儿园49所，在245所农村小学、教学点增设附属幼儿园。深入实施农村义务教育阶段学生营养改善计划，符合条件的学生全部受益。4.强基惠民。修建通村水泥路近1000公里，150个建制村受益。实施水库除险加固61座、中小河流治理工程20条，解决饮水不安全人口32万人。5.安居惠民。新增公共租赁住房6400套，新建经济适用住房100套、棚户区改造500套，新增租赁补贴发放300户。6.土地整治惠民。实施坡耕地改造1.16万亩，治理小流域水土流失76平方公里，新增和恢复农田灌溉3.6万亩，改善灌溉12万亩。7.农补惠民。全面落实农作物良种补贴，农资综合补贴、农机购置补贴等各项惠民补贴政策。8.生态惠民。植树造林40万亩，综合治理岩溶511平方公里，治理石漠化222平方公里。9.文体惠民。完成自治区下达的村级公共服务中心和村村通广播电视、乡镇无线发射台站新建任务。抓好乡镇、村级农民体育健身工程建设。10.计生惠民。计生奖励扶助政策兑现率达100%、诚信计生村依法生育育龄妇女参与率达85%以上、国家免费孕前优生健康检查目标人群覆盖率达86%以上，扶持农村计生家庭小额贴息贷款650户以上，办理农村计生家庭爱心保险2.7万户。

三、加强政府自身建设

我们必须切实转变政府职能，创新行政管理方式，增强政府公信力和执行力，做到为人民服务、对人民负责、受人民监督、让人民满意。

建设依法行政的法治政府。依法履行好公共服务、市场监管、社会管理、环境保护的职能，完善执法程序，提高执法水平。自觉接受人大及其常委会监督和政协民主监督，高度重视公众监督和媒体舆论监督，广泛听取各民主党派、工商联、人民团体和无党派人士的意见。推进网络问政工作制度化、常态化，进一步发挥网络倾听民声、凝聚民智、服务民生的作用。

建设执政为民的服务政府。以开展党的群众路线教育实践活动为契机，坚持把为民服务贯穿于政府工作的全过程。加强公共设施建设，创新体制，改进方式，为群众提供更多更好的公共服务。坚持改进会风、文风，严格控制和规范各种检查、评比、达标活动，减少不必要的应酬和事务性活动。转变工作作风，力戒“两无三不四先”现象，把求真务实的要求落实到政府工作的各个环节，做到一心一意谋发展，集中精力抓落实。

建设严格自律的廉洁政府。认真落实“八项规定”，坚决遏制“四风”现象，艰苦奋斗，勤俭节约，把有限财力更多用于民生改善。进一步健全惩防体系，从源头上预防腐败，从机制上遏制腐败，提高对重点领域、关键部位、特殊岗位的监管水平。全面落实党风廉政建设责任制，强化审计监督和行政监察，严格执行廉洁从政有关规定，严肃查办违纪违法案件，努力实现干部清正、政府清廉、政治清明。

各位代表，新的目标催人奋进，让我们在市委的坚强领导下，以更加高昂的斗志、饱满的热情和务实的作风，攻坚克难，开拓创新，埋头苦干，为加快富民强市新跨越，建设幸福新河池而奋斗！
